# Digital Mindfulness App Utilization and Perceived Stress Among Caregivers of Persons Living With Dementia

**DOI:** 10.1093/geroni/igaf122.2178

**Published:** 2025-12-31

**Authors:** Michael P Williams, Morgan Seward, Elizabeth M Allen, Raquel G Tatar, Jennifer Huberty, Ana-Maria Vranceanu, Evan Plys

**Affiliations:** Massachusetts General Hospital, Boston, Massachusetts, United States; Massachusetts General Hospital, Boston, Massachusetts, United States; Massachusetts General Hospital, Boston, Massachusetts, United States; Healthy Minds Innovation, Madison, Wisconsin, United States; Fit Minded Inc., Phoenix, Arizona, United States; Massachusetts General Hospital, Boston, Massachusetts, United States; Massachusetts General Hospital, Boston, Massachusetts, United States

## Abstract

Mindfulness digital health interventions (DHIs) can reduce stress among caregivers of persons living with dementia (PLWD). Little is known about how mindfulness DHI utilization relates to stress reduction. This study is a secondary analysis of a feasibility pilot RCT of Healthy Minds Program for Caregivers (HMP-C), a mindfulness stress-reduction DHI for caregivers of PLWD. Forty-seven participants randomized to the intervention condition (HMP-C) in the primary trial were included the secondary analysis. Participants were instructed to use HMP-C for a minimum of 52.5 minutes per week. We describe HMP-C utilization and the association between HMP-C usage and reductions in perceived stress (PSS-10) between baseline and post-test using linear and logistic regression. Median usage of HMP-C was 30 minutes per week (IQR = 30; Range= 1-110). Every 10 additional minutes of HMP-C usage per week was associated with a 1.2 (CI = 0.4,1.9) point decrease in perceived stress. Using HMP-C for 52.5 minutes or more was associated with an 8-point reduction in stress (CI = 3.2,13.2). These findings suggest that utilization of HMP-C is related to reductions in perceived stress. However, there was significant variability in utilization, with few caregivers sufficiently using HMP-C to see clinical reductions in perceived stress. Future research is needed to explore strategies for improving utilization among low-usage caregivers and to identify the optimal balance of feasibility and clinical benefit for mindfulness DHIs among caregivers.

